# Case report: Distinct neurologic manifestation and cytokine profile of a child with COVID-19-associated acute fulminant encephalitis

**DOI:** 10.3389/fmed.2023.1209656

**Published:** 2023-06-13

**Authors:** Yu-Ming Chang, Cheng-Han Chen, Jieh-Neng Wang, Chao-Min Cheng, Yi-Fang Tu, Ching-Fen Shen

**Affiliations:** ^1^Department of Pediatrics, National Cheng Kung University Hospital, College of Medicine, National Cheng Kung University, Tainan, Taiwan; ^2^Institute of Biomedical Engineering, National Tsing Hua University, Hsinchu, Taiwan; ^3^Department of Emergency Medicine, Taipei Veterans General Hospital, Taipei, Taiwan; ^4^School of Medicine, National Yang Ming Chiao Tung University, Taipei, Taiwan; ^5^Institute of Clinical Medicine, College of Medicine, National Cheng Kung University, Tainan, Taiwan

**Keywords:** COVID-19, SARS-CoV-2, acute fulminant cerebral edema, encephalitis, cytokine, cerebral edema

## Abstract

The neurologic manifestations of coronavirus disease 2019 (COVID-19) may range from mild symptoms such as headache or confusion to profound encephalopathy with variable outcomes and sequelae. Here, we reported a case of fatal COVID-19-associated encephalitis with acute fulminant cerebral edema, presenting first with visual hallucination and then a rapid progression into comatose status in a few hours. Serial brain computed tomography depicted cerebral edematous changes from bilateral ventral temporal lobe to the whole brain leading to brain herniation. Multiple cytokines in serum and cerebrospinal fluid (CSF) were increased, with a more prominent rise in the CSF. Therefore, we postulated a hypothesis regarding the mechanism of this fulminant encephalitis that the SARS-CoV-2 virus attacked ventral temporal lobes initially, triggered a severe cytokine storm, and then led to subsequent disruption of the blood-brain barrier, diffuse brain edema, and brain herniation. The trend of cytokine profiles over time may aid in diagnosing and evaluating the severity and prognosis of COVID-19-associated encephalitis.

## Introduction

The coronavirus disease 2019 (COVID-19), starting from a national outbreak in Wuhan, China, rapidly became a pandemic and caused huge disease burdens and mortalities worldwide. While respiratory symptoms are the most common clinical presentations, neurologic symptoms such as confusion, agitation, or seizure are also common (1). The neurologic manifestations could range from self-limited symptoms to fatal presentations such as fulminant encephalitis. Here, we report a case of severe COVID-19-associated encephalitis with acute fulminant cerebral edema, mainly to address the distinct clinical presentation and cytokines profile.

## Case description

This 10-year-old previously healthy and COVID-19 naive boy who had not received vaccination for SARS-CoV-2 presented to our emergency department (ED) with a new-onset seizure and conscious disturbance. He had intermittent fever, dizziness, and emesis since the day before presenting to ED. His family members got fever and respiratory symptoms and were diagnosed with COVID-19 a few days before. A few hours before arriving at ED, he claimed he saw a monster in the bathroom and on his family member's shoulders and was terrified. Then, he developed upward gazing, generalized clonic convulsion, and impaired awareness lasting 5 min. Another two similar seizure episodes followed within a half hour, and he failed to regain alertness entirely after the last episode. Throughout the course, there was no involuntary movement, smell or taste change, focal weakness or sensory change. He was taken to our ED for further evaluation and management.

At arrival, the patient was alert but had decreased awareness. He was oriented to place, people but not to time, and his response was slower than his usual status. The temperature was 40.1°C, the blood pressure 105/50 mm Hg, the pulse 130 beats per min, the respiratory rate 22 breaths per min, and the oxygen saturation 98% in ambient air. The pupils were equal in size and reactive to light stimuli. Extraocular movements were intact without nystagmus or involuntary eye movements. Muscle strength was symmetrically full, and deep tendon reflexes were 2+ throughout with flexor plantar reflexes. The neck was supple, and Brudzinski and Kernig signs were negative. The result of a finger–nose–finger test was normal, and the performance of tandem gait was steady without deviation. The remaining physical examination was normal.

**Figure 1:**
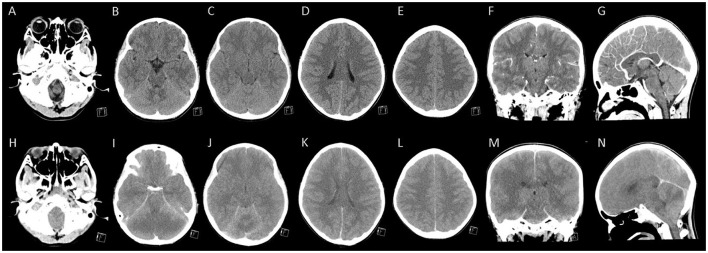
The brain computed tomography of the patient. **(A–G)** Represented contrast-enhanced computed tomography (CT) images performed 4 h after admission showed mild cortical swelling of bilateral ventral temporal lobes that slightly pushed into tentorial incisura. In contrast, the gray-white matter differentiation was preserved in fronto-pareital lobes. **(H–N)** Represented contrast-enhanced CT images performed 16 h after admission (6 h after cardiopulmonary cerebral resuscitation) showed diffuse cerebral and cerebellar swelling and bilateral uncal herniation with the inferior displaced brainstem.

Computed tomography (CT) of the head with contrast medium (Figures 1A–G), performed 4 h after arrival, depicted bilateral mild cortical swelling, especially bilateral ventral temporal lobes, slightly pushing into tentorial incisura, indicating possible acute encephalitis. The laboratory data showed elevated alanine aminotransferase (91 U/L), aspartate aminotransferase (210 U/L), pro-calcitonin (11.85 ng/mL), and interleukin-6 (IL-6) level [0.271 ng/mL, by IL-6 test strip combined with a spectrum-based optical reader (2, 3)]. The cerebrospinal fluid (CSF) analysis later showed no pleocytosis, but a significantly high total protein (387 mg/dL) and interleukin-6 (IL-6) (5.5 ng/mL, by IL-6 test strip) level. He was positive for COVID-19 by reverse transcriptase polymerase chain reactive (PCR) on a nasopharyngeal swab, but the virus was not detected in the CSF by PCR. Other laboratory data was presented in Supplementary Table.

Under the diagnosis of COVID-19-associated acute encephalitis, mannitol (0.5 g per kilogram) was given to reduce intracranial pressure and methylprednisolone pulse therapy (30 mg per kilogram) for anti-inflammation. Initially, this patient could still obey simple commands and responded slowly with few verbal outputs. However, he developed junctional tachycardia, then ventricular tachycardia and soon progressed to asystole 10 h after his arrival. After 15 min cardiopulmonary cerebral resuscitation (CPCR), the patient had a return of spontaneous circulation (ROSC) but became unresponsive, had no spontaneous breathing, and fixed dilated pupils since then. In considering COVID-19-related fulminant encephalitis, intravenous remdesivir (at doses of 5 mg per kilogram on the first day and 2.5 mg per kilogram on the subsequent days), tocilizumab (with a single dose of 12 mg per kilogram), and immunoglobulin (at a dose of 2 grams per kilogram) were administered successively. After ROSC, he was treated with therapeutic hypothermia at a target temperature of 33.5°C during the subsequent 3 days. Six hours after CPCR, another head CT (Figures 1H–N) showed diffuse cerebral and cerebellar swelling and bilateral uncal herniation with the inferior displaced brainstem. Mannitol was administered at a dose of 0.5 g per kilogram every 6 h.

**Figure 2:**
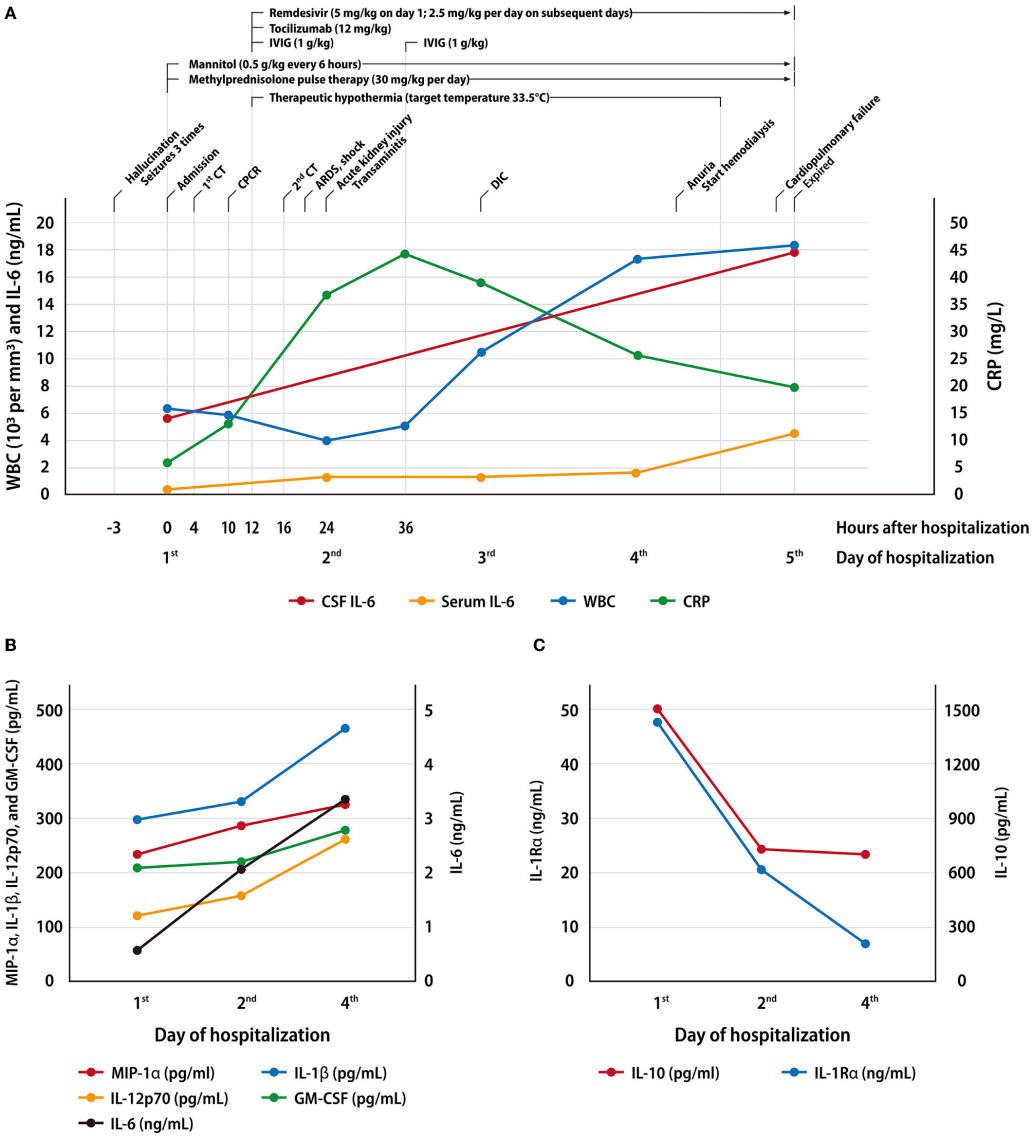
Serial biochemical data and cytokines profile in serum and cerebrospinal fluid. **(A)** The clinical course and the dynamic changes in inflammatory markers of this patient. **(B, C)**. The serum cytokine profile that showed an escalating trend **(B)** and a falling trend **(C)**.

Despite these therapeutic measures, in the subsequent 4 days, the patient remained unresponsive with fixed dilated pupils and had unstable hemodynamic status with systolic hypotension, central diabetes insipidus, and acute kidney injury with anuria. Laboratory examination showed hyperlactatemia, elevated serum creatinine level, transaminitis, hyperferritinemia and coagulopathy (Supplementary Table). Due to the progressive multi-organ failure that occurred in an order of acute respiratory distress syndrome, circulatory shock, hepatic dysfunction, acute renal failure, disseminated intravascular coagulation, and finally cardiac failure complicated by sustained ventricular tachycardia, he passed away on the morning of the fifth day of admission. The PCR for SARS-CoV-2 was negative from a post-mortem tissue biopsy of the lung, heart, liver, spleen, and kidney. The CSF obtained soon after death was negative for SARS-CoV-2, but still has a very high IL-6 level (17.978 ng/mL, by IL-6 test strip). The clinical course and the dynamic changes in inflammatory markers were summarized in Figure 2A. Multiple cytokines were tested from serum and CSF using a cytokine array, Multiplex ELISA Kit For Human Cytokine Release Syndrome (16-Plex) (Bosterbio, Pleasanton, California, USA). The results, presented in Table 1, showed an increase in multiple cytokines including GM-CSF, IL-1β, IL-10, IL-12p70, MIP-1α, IL-2Rα, IL-1Rα, IL-6, IL-8, and MCP-1 from both serum and CSF, with a more prominent rise in the CSF than in the serum. The trend of the cytokines over time is presented in Figures 2B, C.

**Table 1:** Serial serum cytokine profile of this patient and comparison with other COVID-19 cases.

	**IL-1β (pg/ml)**	**IL-1Rα (pg/ml)**	**IL-2Rα (pg/ml)**	**IL-6 (pg/ml)**	**IL-8 (pg/ml)**	**IL-10 (pg/ml)**	**IL-12p70 (pg/ml)**	**GM-CSF (pg/ml)**	**MIP-1α (pg/ml)**	**MCP-1 (pg/ml)**	**TNF-α (pg/ml)**
**Serum**											
**Index case**											
Day 1	298.28	47,638.82	3,494.52	441.19	271.18	1,509.81	125.11	208.98	233.57	1,003.90	77.86
Day 4	330.72	20,559.41	2,573.26	2,190.71	390.22	726.45	157.95	220.76	288.80	1,494.50	139.81
Day 5 (the day of death)	466.76	6,691.84	3,332.99	3,407.35	336.68	696.26	261.60	277.81	326.87	988.71	93.03
**Control case**											
Control case 1	291.91	1,022.85	841.20	19.33	91.45	91.45	112.09	145.55	114.16	490.30	23.31
Control case 2	255.00	7,795.38	1,226.23	102.25	100.36	100.36	84.21	164.90	213.36	832.00	50.92
Control case 3	205.20	904.80	2,427.44	19.46	103.32	103.32	75.99	173.30	226.61	829.37	27.15
**CSF**											
Index case (Day 5; post-mortum)	307.81	23,235.94	1,903.79	4,575.04	24,403.47	395.31	168.46	228.97	389.83	20,193.71	39.65
Control case 1	64.21	83.52	368.76	7.65	13.15	39.09	65.04	104.95	Low	273.94	Low
Control case 4	84.32	434.58	528.05	12.17	30.50	53.34	90.70	139.47	50.13	977.79	9.53

Clinical information of the control cases.

Control case 1, 10-year-old boy, COVID-19 with encephalitis.

Control case 2, 9-year-old boy, COVID-19 with bronchopneumonia.

Control case 3, 9-year-old boy, COVID-19 with acute myositis.

Control case 4, 1-year-6-month-old boy, COVID-19 with myoclonic jerk.

Other pathogens survey included human herpesvirus 6, cytomegalovirus, herpes simplex virus, Epstein-Barr virus, hepatitis B and C, Mycoplasma pneumoniae, multiplex PCR using BioFire FilmArray respiratory panel from throat swab, and BioFire FilmArray meningitis/encephalitis panel (BioFire Diagnostics, Salt Lake City, Utah, USA) from CSF. The FilmArray respiratory panel detects pathogens, including adenovirus, human rhinovirus/enterovirus, influenza virus A (A, A/H1, A/H1-2009, A/H3), influenza virus B, respiratory syncytial virus, parainfluenza viruses 1-4, human metapneumovirus, Coronavirus 229E, HKU1, OC43 and NL63, SARS-CoV-2, Chlamydia pneumoniae, Bordetella pertussis, Bordetella parapertussis, and Mycoplasma pneumoniae, and the FilmArray meningitis/encephalitis panel detects pathogen including *Escherichia coli* K1, *Haemophilus influenza, Listeria monocytogenes, Neisseria meningitides, Streptococcus agalactiae, Streptococcus pneumoniae*, cytomegalovirus, enterovirus, herpes simplex virus 1 and 2, human herpesvirus 6, human parechovirus, varicella zoster virus, *Cryptococcus neoformans*/*gattii*. All these results were negative except SARS-CoV-2 detected from the FilmArray respiratory panel.

## Discussion

We reported a case of fulminant COVID-19-associated encephalitis that is distinct in its extraordinary rapid progression of brain edema within hours and the initial presentation with visual hallucination. We performed a thorough cytokines investigation among serum and CSF to provide prognostic prediction and treatment guidance.

Around one-fourth of children with COVID-19 had neurologic involvement (4). The reported neurologic symptoms of COVID-19 are disorientation/confusion, loss of consciousness, headache, and seizure predominantly. Only a small portion of patients have hallucinations (5, 6). One-tenth of those with neurologic involvement may develop life-threatening conditions, including acute encephalopathy (4). Among those with COVID-19-associated acute encephalopathy, acute fulminant cerebral edema is a recently recognized phenotype and comprises around 2% of them. Fever, seizures, coma, and Asian/Pacific Islander are significant risk factors in children with COVID-19-associated acute encephalopathy, and mortality is significantly higher with this fulminant phenotype (5, 6).

The first neurological manifestation of our patient was a visual hallucination (a monster) with a fear sensation. The initial corresponding brain CT showed trivial cortical swelling of the bilateral ventral temporal lobe. Previous studies examining patients with visual hallucinations by functional magnetic resonance imaging had demonstrated that ventral temporal lobes are associated with hallucinations of costumed figures (7). In addition, due to its proximity to the amygdala, the hallucinations are often related to unpleasant experiences and emotions (7). This evidence supported that the initial preference of the SARS-CoV-2 virus in the central nervous system might be the ventral temporal lobes and amygdala.

The pathogenic mechanisms of COVID-19-associated acute encephalopathy are still uncertain. Up to now, there are two hypotheses about the mechanism of brain damage, one being a direct invasion of the virus and the other being a severe inflammatory process caused by an extensive release of pro-inflammatory cytokines. The excessive pro-inflammatory response theory was supported by the evidence of significant increases in pro-inflammatory cytokines without detecting SARS-CoV-2 virus in CSF (1). This result is consistent with another severe virus-associated encephalopathy, acute necrotizing encephalopathy, in which a significant elevation of IL-6 and other cytokines is detected among CSF without direct identification of viral particles (8). These pro-inflammatory cytokines, in a hypothesis, cause endothelial dysfunction and cytokine storms that further damage the blood-brain barrier (BBB), resulting in the entry of these cytokines into brain parenchyma and the subsequent insults of neurons (1). Hypercytokinemia induces proteolytic destruction of BBB through trypsin and the activation of matrix metalloprotease-9, which causes an increase in vascular permeability resulting in brain edema (9). A considerably higher serum and CSF cytokine level was observed in our patient than in the control group, which might indicate that the higher cytokine level may link to a greater probability of developing cerebral edema. Most interestingly, we observed a more prominent rise of cytokines in the CSF than in serum in our patient, suggesting that the primary site of inflammation and release of cytokines might be the brain itself. Some possible mechanisms have been proposed that SARS-CoV-2 virus might be direct invasion through the olfactory bulb and the cribriform plate of ethmoid bone (10), or a route similar to enteroviral rhombencephalitis that the virus invades the motor neuron of the anterior horn cells of the spinal cord, and then ascends via a neuronal route to the brain (11). Although we did not detect the SARS-CoV-2 virus by PCR among the CSF in this patient, evidence had shown that PCR in the CSF has relatively low sensitivity (1). Studies demonstrate the presence of SARS-CoV-2 virus in the brain tissue on autopsy (12, 13), suggesting the possibility of brain invasion by viral itself.

Previous studies have found that IL-6, IL-1β, IL-8, tumor necrosis factor-alpha (TNF-α), monocyte chemotactic protein-1 (MCP-1), and granulocyte-macrophage colony-stimulating factor (GM-CSF) was highly elevated either in serum or CSF of patients with COVID-19 related encephalitis (1, 14, 15). In addition to the cytokines mentioned above, we also observed a rise in IL-10, IL-12p70, MIP-1α, IL-2Rα, and IL-1Rα among paired samples of CSF and serum (Table 1). IL-6 was deemed as a prognostic factor and potential treatment target of severe COVID-19 cases (16). Among these cytokines tested in serum, we observed an initial peak with a falling trend over time in IL-10 and IL-1Rα, and a rising trend in IL-6, MIP-1α, IL-1β, IL-12p70, and GM-CSF (Figures 2B, C). We propose that the former group might serve as an early diagnostic indicator of fulminant cerebral edema due to its rapid peaking and considerably higher level in cases with cerebral edema than in those without cerebral edema among patients with COVID-19-associated encephalitis. Early identification of hypercytokinemia could hint clinicians at a possibly fulminant course. The latter group of cytokines might be regarded as a prognostic factor which, in cases with an increasing trend, indicates a poor outcome. Although these cytokines are not tissue-specific, severe neurologic disease should be considered if the level of hypercytokinemia is higher in the CSF than in the serum, as seen in our case. This hypothesis needs to be validated further.

In our case, it is likely that the SARS-CoV-2 virus attacked ventral lobes initially, triggered a severe cytokine storm, and then led to subsequent cascades, including disruption of the blood-brain barrier, fulminant brain edema, and brain herniation. The strength of our study lies in the comprehensive dynamics of the pro-inflammatory (mainly IL-1β, IL-6, IL-8, TNF-α) and anti-inflammatory (IL-10) cytokines over time and their comparison with non-severe COVID-19 cases as supporting evidence. The interplay of these cytokines during disease progression might help to elicit underlying immune mechanism and serves as an early biomarker. Nevertheless, the limitation is that the magnetic resonance imaging scan is not available in the presented case due to his rapidly progressing course and critical status. Future study with larger cases population is needed to validate this result.

In conclusion, we report a case of a boy with severe fatal COVID-19-associated encephalitis complicated by acute fulminant cerebral edema. The distinct visual hallucination and its corresponding ventral temporal lobes swelling on brain imaging might be a clue to the primary site of COVID-19-associated encephalitis that subsequently spread to the whole brain. The trend of serum cytokine levels may serve as diagnostic and prognostic biomarkers. The relatively high IL-6 level in the CSF hints that the primary inflammation site is the brain itself, leading to fulminant brain edema.

## Data availability statement

The original contributions presented in the study are included in the article/Supplementary material, further inquiries can be directed to the corresponding authors.

## Ethics statement

The Institutional Review Board of National Cheng Kung University Hospital reviewed and approved the study protocol (IRB No. B-EC-112-004). Written informed consent to participate in this study was provided by the participants' legal guardian. Written informed consent was obtained from the participant's legal guardian for the publication of any potentially identifiable images or data included in this article.

## Author contributions

Y-MC, C-FS, and Y-FT were responsible for conceptualization. J-NW, C-MC, C-HC, and C-FS were responsible for data curation. Y-MC was responsible for the original drafting. Y-FT and C-FS for reviewing and editing. All authors have read and agreed to the published version of the manuscript.
