# Supplementary material for: Case report: Distinct neurologic manifestation and cytokine profile of a child with COVID-19-associated acute fulminant encephalitis
Source: Front Med (Lausanne). 2023 Jun 13;10:1209656. doi: 10.3389/fmed.2023.1209656 (PMC10299828; doi:10.3389/fmed.2023.1209656)
Supplement: Supplementary file 1 [file Data_Sheet_1.PDF]

Supplementary Table. The laboratory data of this patient.

Variable	Reference range	First presentation, emergency department	CSF (the day of admission)	After CPCR (the day of admission)	The fourth day of admission
Hemoglobin (g/dL)	10.2-14.5	12.5		10.9	9.9
Platelet (1000 per mm3)	151-332	229		206	129
White cell count (per mm3)	4000-11000	7600		7200	17600
Sodium (mmol/L)	136-145	141		152	158
Potassium (mmol/L)	3.5-5.1	2.7		3.0	4.5
Calcium (mg/dL)	8.6-10.0	9.0		8.8	8.4
Glucose (mg/dL)	60-99	80		167	215
Creatinine (mg/dL)	0.4-1.0	0.69		1.25	2.57
ALT (U/L)	<50	91		379	4730
AST (U/L)	<50	201		595	11852
C-reactive protein (mg/L)	<8.0	5.4		12.7	25.6
Procalcitonin (ng/mL)	<0.05	11.85			
Ferritin (ng/mL)	30-400				4353
Lactate (mmol/L)	0.5-2.2	3.5		12.6	4.1
Ammonia (ummol/L)	16-60	48		25	24
PT (INR)	0.8-1.2			2.0	2.1
APTT (secs)	29.3-40.1			47.2	38.7
Turbidity			Clear		
White cell count (/mm3)	0-10		5		
Red cell count (/mm3)	0		5		
Glucose (mg/dL)	40-70		64		
Total protein (mg/dL)	12-60		387		
Lactate (mmol/L)	0.7-2.1		2.7		
Interleukin 6 (ng/mL)	<0.007	0.271	5.549		
